# Demecology in the Cambrian: synchronized molting in arthropods from the Burgess Shale

**DOI:** 10.1186/1741-7007-11-64

**Published:** 2013-05-30

**Authors:** Joachim T Haug, Jean-Bernard Caron, Carolin Haug

**Affiliations:** 1 Zoological Institute and Museum, Department of Cytology and Evolutionary Biology, Soldmannstrasse 23, 17487 Greifswald, Germany; 2 Department of Natural History - Palaeobiology, Royal Ontario Museum, 100 Queen's Park, Toronto, Ontario, M5S 2C6, Canada; 3 University of Toronto, Department of Ecology and Evolutionary Biology, 25 Willcocks Street, Toronto, Ontario, M5S 3B2, Canada

**Keywords:** Burgess Shale, Cambrian bioradiation, ‘Cambrian explosion’, Demecology, Molting, Nursery habitats

## Abstract

**Background:**

The Burgess Shale is well known for its preservation of a diverse soft-bodied biota dating from the Cambrian period (Series 3, Stage 5). While previous paleoecological studies have focused on particular species (autecology) or entire paleocommunities (synecology), studies on the ecology of populations (demecology) of Burgess Shale organisms have remained mainly anecdotal.

**Results:**

Here, we present evidence for mass molting events in two unrelated arthropods from the Burgess Shale Walcott Quarry, *Canadaspis perfecta* and a megacheiran referred to as *Alalcomenaeus* sp.

**Conclusions:**

These findings suggest that the triggers for such supposed synchronized molting appeared early on during the Cambrian radiation, and synchronized molting in the Cambrian may have had similar functions in the past as it does today. In addition, the finding of numerous juvenile *Alalcomenaeus* sp. molts associated with the putative alga *Dictyophycus* suggests a possible nursery habitat. In this nursery habitat a population of this animal might have found a more protected environment in which to spend critical developmental phases, as do many modern species today.

## Background

The incompleteness of the fossil record makes reconstructing animal ecosystems of the past, a particularly challenging task. Fortunately, a few exceptional sites, generally referred to as fossil *Lagerstätten*, preserve far more paleoecological information than do normal fossil deposits. Arguably the best-known *Konservat-Lagerstätte* is the famous middle Cambrian (Series 3, Stage 5; ca. 505 million years old) Burgess Shale in British Columbia, Canada. This deposit is famous for its exquisite preservation of soft-bodied animals, and provides critical clues to the structure and functioning of animal communities in the aftermath of the Cambrian bioradiation (often called the ‘Cambrian explosion’).

Our knowledge of the Burgess Shale community is mostly based on autecological studies involving reconstructions of the fossilized organisms’ functional morphology, as well as detailed comparative anatomical studies (for example, [1-3]). However, autecological reconstructions can serve as a basis for synecological consideration, e.g., predator-prey interactions [4], and the study of larger community and ecological patterns (for example, [5]). Although paleoecological investigations are often limited to morphological information about the species under investigation, the Burgess Shale also provides evidence of animal activities in the form of trackways [6], other trace fossils [7], and gut contents [8]. Even behavior is sometimes preserved by being “frozen” in time, for example, a specimen of *Marrella splendens* Walcott, 1912 was caught in the act of molting [9], several specimens of *Ottoia prolifica* Walcott, 1911 were preserved while scavenging together on a carcass [8,10], and trilobites have been preserved within the empty tubes of priapulid worms [11]. Such data, although still scarce, add to our knowledge of both individual behavior (autecology) and interactions among coexisting species (synecology). Direct synecological evidence also includes brachiopods attached to sponges [12], but, so far, detailed records of behavior at the population level (that is demecology) in Burgess Shale-type deposits have been limited to the arthropod *Synophalos xynos* (Hou et al., 2009) from the Chengjiang Biota walking in chains [13,14], and the finding of similar food items in the guts of entire populations of *Ottoia prolifica*[8].

Here, we report the finding of hundreds of exuviae of two different arthropods from the Burgess Shale Walcott Quarry. Together, they represent the largest accumulation of soft-bodied fossil exuviae known from Cambrian Burgess Shale-type deposits. The finds indicate synchronized molting behavior for populations of two different species. Possible causes and mechanisms of such coordinated behavior are discussed.

## Methods

All the studied specimens are stored at the Royal Ontario Museum (ROM) in Toronto, Canada. All specimens were collected *in situ* below the base of the original Walcott Quarry (Fossil Ridge, Yoho National Park, Canada) in different stratigraphic intervals interpreted as representing individual burial events preserving community snapshots [5,15]. In this paper, we illustrate several large clusters of specimens of *Canadaspis perfecta* (Walcott, 1912) [16] (for example, ROM 56954 and ROM 62274) collected from level -350 (approximately 350 centimeters below the base of the original Walcott Quarry, see [15]), as well as several megacheiran clusters (ROM 62275) and individual specimens (ROM 57711) collected at level -120. A single specimen of *C. perfecta* (ROM 61119) with gut trace, collected at level -320, is also illustrated for comparison with the molt assemblages. For all slabs, the presence of abundant and diverse soft-bodied organisms representing a range of sizes and belonging to different groups of organisms suggests no significant taphonomic biases, including transport and time averaging [15,17].

**Figure 1:**
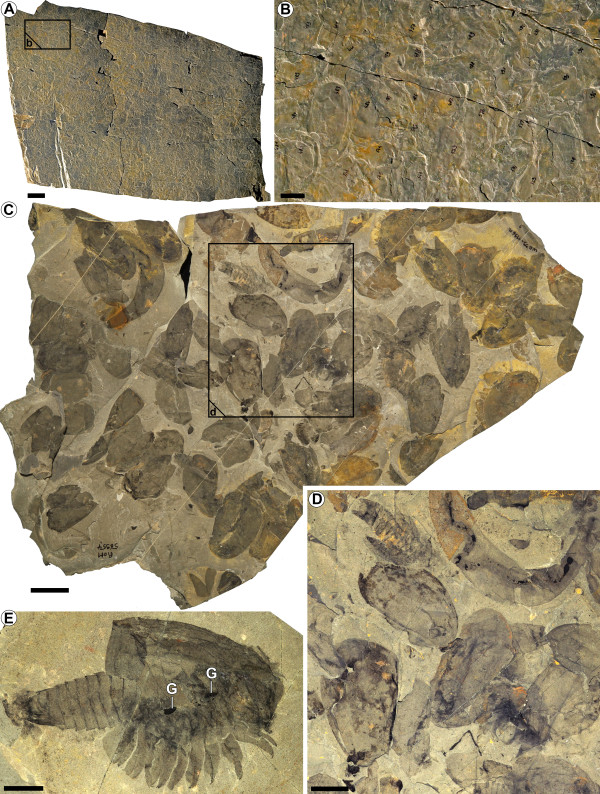
***Canadaspis perfecta *(Walcott, 1912). (A,B) **ROM 62274, supposed synchronized molting (-350 level); **(A) **complete slab; **(B) **close-up image of **(A) **showing dozens of shields. **(C,D) **ROM 56954; **(C) **aggregation of carcasses from the same level (-350); **(D)** close-up image of **(C)**; **(E) **ROM 61119, close-up image of a single carcass showing limbs and evidence of gut **(G) **(-320 level). Scale bars: 10 cm **(A)**; 2 cm **(B,C)**; 1 cm **(D,E)**.

**Figure 2:**
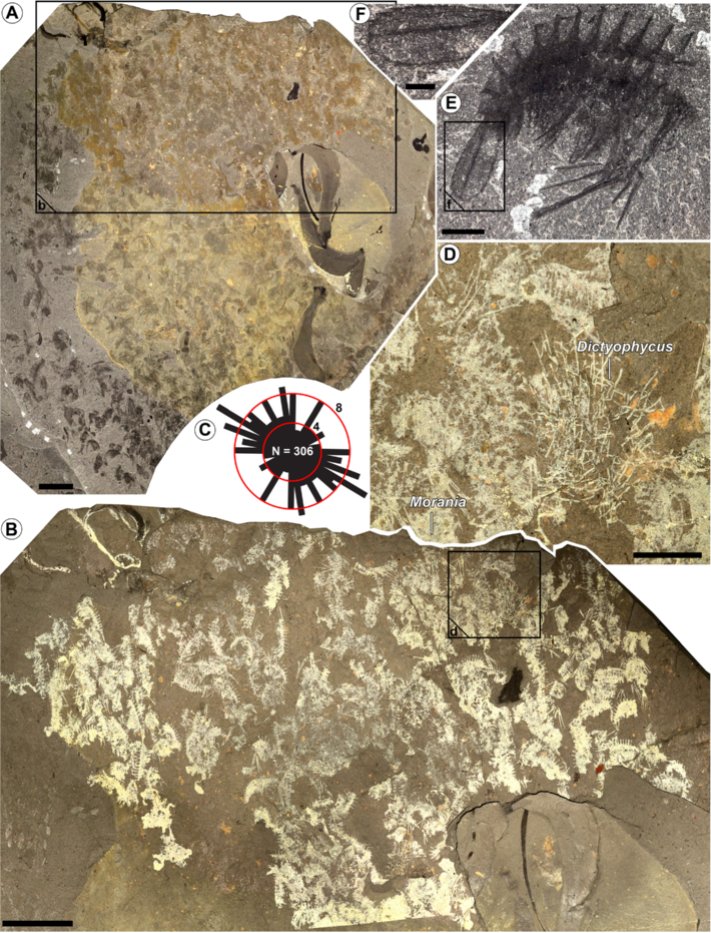
**Supposed synchronized molting of megacheirans. (A-D) **ROM 62275; **(A) **overview of the specimens (cross-polarized light); **(B) **close-up image of **(A)**; **(C)** rose diagram of relative orientation of megacheiran specimens (N = 306); **(D)** close-up image of **(B) **showing associations with *Dictyophycus gracilis* Ruedemann, 1931, a putative alga, and *Morania *spp. Walcott, 1919, a putative cyanobacteria; **(E,F) **ROM 57711, single specimen with well-preserved telson (close up in **(F)**). Scale bars: 2 cm **(A,B)**; 0.5 cm **(D)**; 0.2 cm **(E)**; 0.1 cm **(F)**.

**Figure 3:**
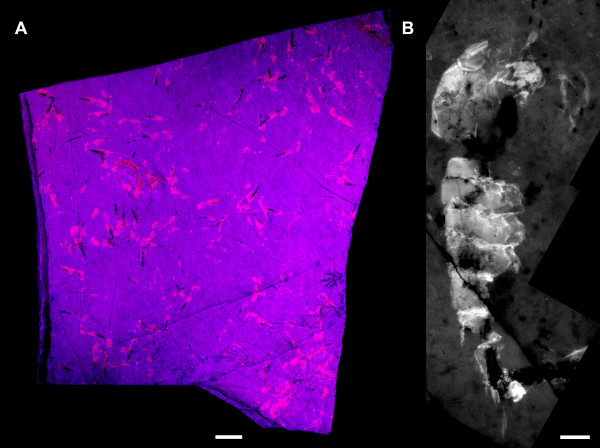
***Anthonema problematicum *Walther, 1904, Upper Jurassic, Solnhofen Lithographic Limestones, Southern Germany. (A)** Mass aggregation of exuviae under macrofluorescence, SMNS 70109, ex coll. Frattigiani, Laichingen; **(B)**, close-up image of single exuvia under microfluorescence conditions. See [18,19] for details on methods. Scale bars: 1 cm **(A)**; 1 mm **(B)**.

Specimens in Figures 1 and 2 were photographed under normal or polarized light with a digital camera. Some close-up images were taken with a Scopetek DCM 510 ocular camera (Hangzhou Scopetek Opto-Electric Co., Hangzhou, China) through a Nikon SMZ 1500 stereomicroscope (Nikon, Tokyo, Japan). Images in Figure 3 were, in most cases, recorded as stacks and fused with CombineZM/ZP (Alan Hadley). More than one stack was recorded for some specimens when the field of view was limited. Each stack of images was fused, and the fused images were then stitched together with Adobe Photoshop CS3 (Adobe Systems, San Jose, CA, USA) or Microsoft Image Composite Editor (Microsoft Corporation, Redmond, WA, USA).

Additionally, a mass aggregation of exuviae of the malacostracan crustacean *Anthonema problematicum* Walther, 1904 from the Upper Jurassic Solnhofen Lithographic Limestones of southern Germany was examined. The slab was collected by R. Frattigiani, Laichingen, and is part of the collection of the Staatliches Museum für Naturkunde Stuttgart under the repository number SMNS 70109. The complete aggregation was documented under macrofluorescence, and a single specimen under microfluorescence conditions (wavelength 546 nm; for details, see [18] and [19]).

## Results

A collected slab about 1 m^2^ in area (ROM 62274, Figure 1A,B) is almost entirely covered in individual shields of the bivalved arthropod *Canadaspis perfecta*. At least 743 specimens are preserved on this surface, of which fewer than 10% show evidence of preservation of the thoracic region. There is no evidence of specimens showing preservation of the gut (Figure 1B). This slab was collected from an area about 45 m^2^ in total, which exhibited a similarly dense association of fossils, suggesting that tens of thousands of specimens were buried along this particular bedding interval, most of which belonged to *C. perfecta*. The specimens are so densely packed that they overlap each other and could represent a mass mortality event. However, most, if not all, of these specimens are identified as exuviae based on their preservation and lack of gut traces (for comparison with known carcasses of *C. perfecta*, see the slab collected from the same layer, and one at a slightly different layer, as shown in Figure 1C-E). Most of the shields appear to be deformed, indicating their softness at the time of preservation. This deformation and poor preservation is not a taphonomic effect, as other organisms preserved on the same slab (ROM 62274), such as some specimens of *Wiwaxia corrugata* Matthew, 1899, *Ottoia prolifica* (often with preserved gut supporting the view that the lack of gut in *C. perfecta* is not an artifact)*,* and up to 35 additional species, are all well preserved and do not show evidence of disassociation or breakage (see [5]). Although the size of the specimens cannot be measured with confidence due to their softness, overlap at burial and diagenetic deformation, as well as post-diagenetic tectonization (which has left many specimens sheered with a glossy surface), all specimens appear to be roughly similar in size.

ROM 62275 and ROM 57711 are covered in fossils of a megacheiran (“short great-appendage”) arthropod (Figure 2). A single slab (0.1 m^2^) contains more than 300 specimens on the same bedding plane (Figure 2A,B) and there is no evidence of preferential orientation of the specimens, suggesting limited or no transport (Figure 2C). The largest specimens are up to 2 cm in length from the posterior margin of the telson to the anterior margin of the first somite. The preservation is quite faint (Figure 2D,E), while other specimens from other species occurring on the same slabs are well preserved. The somite boundaries are preferentially preserved, producing a typical denticulate pattern, suggesting that the tegument covering the segments was very thin and possibly decayed. The trunk appendages are poorly preserved, and the telson is missing in most cases (but see Figure 2E,F). All specimens also appear to lack the head shield (Figure 2E). When the frontal appendages are present, they are often tilted ventrally and at a right angle when seen in a lateral view (Figure 2E). The lack of a cephalic shield, the right angle between the frontal appendages and the trunk, the presence of numerous isolated parts, and the poor preservation of exoskeletons support the hypothesis that the specimens observed in this study are molts and not carcasses. The specimens clearly represent a megacheiran species because of their similar number of segments (11) and great appendage morphology, which features 3 elongated finger-like spines that continue into (possibly) multiannulated feeler-like structures. The specimens seem to differ from all other known megacheiran arthropods from the Burgess Shale, however, in telson shape and ornamentation. The telson shape of this form is elongated and pentagonal, with spines arranged along the entire margin. These can be differentiated into larger and smaller spines. Additionally, the telson has a pronounced keel not known in other megacheirans from the Burgess Shale. Whether these differences represent valid taxonomic differences or whether these individuals can be considered juvenile stages of known megacheirans, such as species of *Alalcomenaeus*, is uncertain. A detailed morphological description of this material and a taxonomic treatment of the species is not part of this study, and following previously published reports [5,15], the material is here referred to as *Alalcomenaeus* sp*.*

## Discussion

### Exuvial nature of the fossils

As mentioned above, the specimens found here in aggregations are interpreted as exuviae. The shields of *Canadaspis perfecta* appear to be slightly deformed, distantly reminiscent of “*Nathorstia transitans*” specimens of *Olenoides serratus* (Rominger, 1887) [20]. *Nathorstia transitans* was originally considered to be a species of a kind of soft-bodied trilobite, but Whittington [20] could demonstrate that these are remains of freshly molted specimens of *Olenoides serratus*.

The exuvial nature of the small megacheirans is also apparent due to their faint preservation in comparison with other specimens on the same slab. In addition, the incompleteness of the fossils, in this case the systematic lack of the head shield and ventral rotation of frontal appendages, are clear indicators of their exuvial nature as previously reported [15]. This state of preservation resembles the “open molt position” of a number of modern decapod crustaceans [21,22], as well as fossil lobsters and other fossil decapods [23,24].

It must be noted that, although the specimens of *Canadaspis perfecta* (ROM 62274) as well as those of *Alalcomenaeus* sp. (ROM 62275, ROM 57711) are interpreted as exuviae, they differ significantly. The specimens of *C. perfecta* almost exclusively feature preserved shields, while the small megacheirans show preservation of most of the body, but always lack the shield. This could indicate that only the more strongly sclerotized parts of the organisms are preserved, but, given that the shield is usually more strongly sclerotized than other parts, it would be expected to be present in the specimens of the megacheiran arthropod. The absence of preserved megacheiran arthropod shields could also be understood as an indication of a two-step molting process: for example, first the shield is molted, and then, later, the remaining body parts. A two-step molting process would explain why there are mostly shields preserved in the *C. perfecta* specimens, while in the *Alalcomenaeus* sp. specimens, everything other than the shield is preserved. Two-step molting is known in extant arthropods, especially in isopods. In isopods, the anterior and posterior body regions are molted separately [25]. This demonstrates that two-step molting is possible in principle. The pattern assumed for the two fossil assemblages described here, that of molting head shields and then molting the remaining body parts, however, is not known from modern arthropods. The inference of such a molting pattern must therefore remain speculative. Nevertheless, this pattern provides a plausible explanation for the observed fossils. A taphonomic explanation is less likely; the fossil assemblages are mixed with specimens of different sizes belonging to different species, and there is no evidence of preferential sorting by size, which could have selectively preserved some parts and not others.

### Synchronized molting: triggers

The presence of many aggregated exuviae of a single species indicates synchronized molting behavior of an entire population or, at least, of a cohesive part of it, that is, of a sub-population. Such coordinated molting behavior is known from a wide variety of extant arthropod groups, including various crustaceans, insects and arachnids.

Synchronization of molting can be triggered by external abiotic factors. Johnson et al. [26] reported synchronized molting in aphids, and interpreted this to be caused by the circadian rhythm. Circadian rhythms have also been suggested to be important for synchronizing molt timing in aquatic organisms, for instance, for larval molts in lobsters [27]. Tarling and Cuzin-Roudy [28], however, discussing the possibility of the influence of external factors, excluded external Zeitgebers (factors influencing timing) from the factors responsible for synchronized molting in krill. They propose active communication among individuals as the main trigger for synchronization.

For terrestrial arthropods, synchronized molting appears particularly often to be actively induced by the organisms themselves through pheromone communication. This has been demonstrated for colonial spiders [29] and group-living collembolans [30], and was also suggested for dermestid beetles [31]. In these examples, however, the groups that synchronize their molting are relatively small. In krill, which congregate in much larger groups, molting is synchronized at the population level [28] with at least 50% of the entire population molting within 48 h [32]. For krill, pheromones may well play an important role, but it remains to be demonstrated; visual communication has also been proposed (see [28] for references).

It is, of course, difficult to infer the means of communication among fossilized individuals. Yet, the fact that a large part of a (sub-) population molted at the same time must be seen as an example of a “smoking gun” (for example, [33]), indicative of communication among individuals or an external stimulus, or both. The most plausible assumption concerning communication would be that it was mediated by pheromones, especially given that hormones coupled to molting are known to be used for communication in modern arthropods. For instance, the sex pheromone of shore crabs is assumed to be a metabolic derivative of the molting process [34], and appears to be also present in other decapods. Sea spiders (Pantopoda) also use molting pheromones as a feeding deterrent for defense against attacking arthropods [35]. It is therefore not unlikely that, even as early as half a billion years ago, arthropods “communicated” via pheromones to synchronize their molts. Recent findings indicate that the brains of Cambrian arthropods were already relatively complex, facilitating quite complex behavior [36,37].

The fossil assemblages described here as possible examples of synchronized molting are also important for understanding the ontogeny of these arthropods, as there is likely an external trigger influencing the timing of molting and, therefore, also growth. The developmental biology of fossil organisms has become an important field of research, known as palaeo-evo-devo [38,39] (see also [40]). The developmental biology of extant organisms has also gone one step further toward understanding which external factors influence ontogeny, under the name of eco-devo [41,42]. The Burgess Shale examples described here could be seen as a first step toward a paleo-eco-devo approach. It is a first indication of how external triggers may have influenced the development of individual fossil arthropods.

### Synchronized molting, function 1: reproduction and predation prevention

Molting in malacostracan crustaceans is often coupled to reproduction and maturation of the ovaries (for example, [43,44] and references therein), as both molting and gametogenesis are controlled by the same hormone [45]. One such example is krill, where synchronized molting behavior has been demonstrated to be coupled to ovary development and spawning [28]. This synchronization of spawning in krill is thought to reduce the predation pressure on the spawning adults and the offspring [46].

The coupling of molting and mating (in this context, mating should be clearly distinguished from spawning, see below) is also known from other extant arthropods, but is restricted to “soft-shell maters,” that is, species that only mate right after molting. Examples can be found in different crustaceans (see [47]), for example, in crabs [34] and isopods [48,49]. This behavior is a highly derived feature, and as far as it is known, it is not linked to synchronized molting involving more than one mating couple. Synchronized molting has been suggested to be coupled to mating for fossil organisms such as trilobites [50,51] and eurypterids [52,53]. In some of these studies, such an assumption appears to be partly based on comparisons with extant xiphosurans, but such comparisons are problematic; while indeed several populations of *Limulus polyphemus* (Linnaeus, 1758) meet in large groups to come ashore and mate (e.g., [54]), this process is not coupled to molting at all. Furthermore, not all populations of *L. polyphemus* mate in large densities, and under low densities the females exhibit monogamous mating behavior [55]. Therefore, synchronized mating is not a behavior generally characterizing the species of extant xiphosurans. It does not appear to be a ground pattern feature of Xiphosura; so, based on phylogenetic reasoning, there is no indication for synchronized mating in eurypterids [53]. Newer evidence indicates that spermatophores were involved in eurypterid mating, so their mating was more similar to that in scorpions and other arachnids than to that in xiphosurans [56]; considering this aspect, mating and spawning was most probably decoupled in eurypterids. Although we cannot completely exclude a coupling of synchronized molting and mating in eurypterids or in trilobites, these data do not provide positive evidence for such a coupling. In both groups, however, the coupling of synchronized molting and spawning, as is known to occur in krill and other crustaceans, could yield a plausible explanation for the observed aggregations of exuviae; this hypothesis could be supported if also eggs and/or larvae are found in these places.

In the examples described in this paper, synchronized molting coupled to spawning is also a possible explanation for the aggregation of *Canadaspis perfecta* exuviae. The specimens are of large size and appear to represent adults exclusively. Thus, it is plausible to assume that a whole population, or sub-population of a seemingly gregarious species actively coordinated molting. The coupling of molting *en masse* to spawning would have reduced the predation pressure not only on individual organisms, but especially on the resulting offspring.

For the case of the small megacheiran fossils, this argument cannot explain the supposed synchronization of the molts. All specimens noted are most likely immature juveniles, much too small to be adults (supposed adults are present in further material still under study), but too large to represent young larvae. Nevertheless, the reduction of predation pressure on animals molting in a group seems to be applicable here as well.

Aside from the coupling of synchronized molting and reproduction, another ecological function has been proposed for the aggregation of exuviae. Kim [57] suggested that group molting among colonial spiders is an adaptation that maintains a similar size among all individuals, which helps to reduce cannibalism during the molting period. This could indeed be an explanation for the case of the supposed synchronized molting in the new megacheiran, as megacheirans are also interpreted as predators [2,3,58].

Mass molting to reduce cannibalism could also be applicable in other cases in which predatory species synchronize their molting, for example, the above-mentioned case of eurypterids, or the occurrence of large masses of mantis shrimps (Stomatopoda) in Carboniferous deposits in Germany [59]. A coupling of cannibalism prevention to reproduction would be plausible in these predatory species; if molting is coupled to reproduction, there should be multiple selective pressures at play to reduce predation on the new young and the molting parents simultaneously, one part of it being the reduction of cannibalism.

Given the multiple selective pressures favoring synchronized molting over uncoordinated molting, it would follow that synchronized molting should be the rule among arthropods, but it is not. Coordination of behavior has several costs. In particular, and most importantly, a communication system, in this case, most likely a pheromone that triggers the behavior, has to evolve. These costs only pay off if the density of individuals is high enough to truly provide real “safety in numbers” during the molt, that is, if synchronized molting provides such a mass of possible prey that the danger to a single individual is significantly reduced. Additionally, if the synchronized molting is communicated via a pheromone, this pheromone could also act as a kairomone to attract predators. The presence of large carcasses of the priapulid *Ottoia prolifica* on the slabs with the megacheiran exuviae could be interpreted as an indication for such an interpretation. Still, given the fact that the evolution of a pheromone coupled to molting appears to be relatively simple and that there are numerous examples among extant species where synchronized molting provides a benefit, such pheromones might have been more widespread among fossil arthropods than currently recognized.

### Synchronized molting, function 2: Burgess Shale fossil associations, possible nursery habitats

Associations of juveniles of marine invertebrates with macrophytes are well documented in many marine environments from temperate to tropical waters. These zones are referred to as nursery or juvenile habitats [60,61]. Macrophytes provide a food supply and support for organisms with complex life histories that require settlement sites during their post-larval and juvenile stages, and offer ideal conditions for animals to molt in a relatively safe environment [61-63]. Experiments on seagrass meadows have shown that predation upon seagrass shrimps is higher on unvegetated bottoms than on vegetated ones, demonstrating the role of macrophytes as predatory refuges as well [64]. Nursery habitats dominated by macrophytes are usually in shallow littoral zones and in relatively protected areas, for example, the nursery habitats of shallow water palinurids usually range from 1 m to 4 m deep, though they can also be found much deeper than 20 m [65]. Algal cover can remain abundant below 200 m in tropical waters [66], and nursery habitats dominated by algae can potentially extend down to the limit of the photic zone. A diverse fauna associated with detached algae has also been discovered in a deeper benthic community in an open shoreline environment, demonstrating that algae play an important role in the recruitment of marine invertebrates even after algal death and transport [67]. Even though macrophytes are crucial to the organization and diversity of modern marine shallow communities [61], the origin and evolutionary significance of nursery areas have never been assessed. One potential reason for this disinterest is that nursery areas, which are composed of juveniles of many invertebrates, have a very low potential of preservation: many marine invertebrates, especially juveniles, are usually composed of soft tissues that do not preserve well in the fossil record [68]. The Burgess Shale megacheirans are often associated with *Dictyophycus gracilis* Ruedemann, 1931, a putative algae (Figure 2D; [69]), and are often entangled with fragments of *Morania spp*. Walcott, 1919, a group of putative cyanobacteria (Figure 2D; [70,71]). These organisms might have provided suitable substrates for the megacheirans to molt. While it is difficult to be positive about this implied relationship, the Burgess Shale might represent one of the oldest examples of a nursery habitat preserved in the fossil record (see [72] for a possibly older example).

### Synchronized molting: fossil record

The cases described in this paper represent the oldest known occurrences of possible synchronized molting in non-calcified arthropods. The only older supposed example was described from trilobites from the early Cambrian of South Australia [72]. The next youngest examples are the supposedly synchronized molts of Ordovician trilobites [51] and of eurypterids in the Silurian [52,53]. Trilobites from the Devonian are also known to occur as aggregated exuviae, although in smaller groups [50]. Another example is that of fossil mantis shrimps from the Carboniferous [59]. In the latter, however, the exuviae appear to co-occur with carcasses. From the Jurassic, the supposed mysid shrimp *Anthonema problematicum* has been found in (calculated) abundances of up to 30,000 specimens per m^2^, all of them representing exuviae (Figure 3A,B; [73]).

As these examples show, there are several cases of supposed synchronous molting events in the fossil record of representatives of modern groups. Trilobites, however, are a counterexample, as they are derivatives of the evolutionary lineage towards Crustacea *sensu lato* (= Mandibulata plus close relatives) [74-76]. Both cases described in this paper also represent species that branched off their evolutionary lineages before the crown group of, possibly, Euarthropoda and Chelicerata, respectively. This indicates that early forms of arthropods also appear to have performed complex interactions at population levels in similar ways to those known from various extant in-group representatives.

## Conclusions

Whether the supposed synchronized molting events described here were indeed coupled to complex chemical communications among individuals of the same population must remain speculative. This is, after all, the first case described in which supposed synchronized molting has been found in two separate species in the same *Lagerstätte*. It may also be likely that different ecological functions triggered the two cases described here. While synchronized molting might have also occurred in other Burgess Shale species, in particular those that occur in large clusters of specimens (for example, in the bradoriid *Kunmingella burgessensis*), the fossil examples provided in this study are the clearest cases found so far.

The Burgess Shale biota once more contributes exciting new insights into Cambrian life. The new findings presented here and the resulting discussion can be seen as first steps into a palaeo-eco-devo approach. This approach, combining population-level ecology (demecology) and coordinated behavior, again, promises more surprises in the future.

## Competing interests

The authors declare that they have no competing interest.

## Authors’ contributions

All authors documented the specimens, processed the images, drafted the manuscript and read and approved it.
